# FSIP2 can serve as a predictive biomarker for Clear Cell Renal Cell Carcinoma prognosis

**DOI:** 10.7150/ijms.48971

**Published:** 2020-10-08

**Authors:** Yixiao Zhang, Xudong Zhu, Xinbo Qiao, Lisha Sun, Ye Tian, Yongliang Yang, Yuhong Zhao, Caigang Liu

**Affiliations:** 1 Department of Oncology, Shengjing Hospital of China Medical University, Shenyang, Liaoning Province, 110004, China.; 2 College of Medicine and Biological Information Engineering, Northeastern University, Shenyang, Liaoning Province, 110169, China.; 3 Center for Molecular Medicine, School of Life Science and Biotechnology, Dalian University of Technology, Dalian, Liaoning Province, 116023, China.; 4 Department of Clinical Epidemiology, Shengjing Hospital of China Medical University, Shenyang, Liaoning Province, 110004, China.

**Keywords:** FSIP2, ccRCC, prognosis, predictive biomarker

## Abstract

**Purpose:** To characterize the role of fibrous sheath interacting protein 2 (FSIP2) in the survival outcomes and prognosis of clear cell renal cell carcinoma (ccRCC) patients, which is currently not well understood.

**Methods:** The Oncomine and CCLE databases were used to investigate the differential expression of FSIP2 in ccRCC versus other cancer types. Levels of FSIP2 in 85 ccRCC patients were assessed by immunohistochemical analysis; clinicopathological features related to FSIP2 expression were examined in these patients finally, disease-free survival and overall survival were estimated by survival analysis to elucidate the impact of FSIP2 expression in ccRCC patients.

**Results:** Analysis using the Oncomine database revealed significant upregulation of the FSIP2 gene in papillary RCC, compared to that in normal tissues. Additionally, FSIP2 expression was found to be significantly associated with abnormal platelet count, positive distant metastasis, and death as the incidence of distant metastasis and death were higher in patients with FSIP2 expression compared to those without FSIP2 expression. Survival analysis revealed that FSIP2 expression was significantly related to shorter disease-free survival and overall survival. Meanwhile, patients with FSIP2 expression had worse prognosis than those without FSIP2 expression.

**Conclusions:** FSIP2 expression is associated with poor survival outcomes and poor prognosis in ccRCC patients. FSIP2 may therefore serve as a potential predictive biomarker of ccRCC prognosis.

## Introduction

Renal cell carcinoma (RCC) accounts for approximately 5% and 3% of all cancers among men and women, respectively, with approximately 70,000 cases reported annually in the USA 1. Radical nephrectomy is the primary treatment method for RCC as radiotherapy and chemotherapy are not associated with improved survival outcomes 2. Although the diagnosis and treatment strategies for RCC have improved, developments related to prognosis and survival outcomes remain poor 3 owing to recurrence and metastasis 4. However, the molecular mechanisms underlying the recurrence and metastasis of RCC are poorly understood 5. Thus, exploring these mechanisms and identifying associated molecules may help in the discovery of novel therapeutic targets, thereby improving the survival outcomes of RCC patients and reducing RCC-associated mortality.

Fibrous sheath interacting protein 2 (FSIP2), a member of the testis antigen family, is a spermatogenic cell-specific protein that is reportedly associated with spermatogenesis 6. Whole-exome sequencing revealed that mutations in the FSIP2 gene are a recurrent cause of morphological abnormality in sperm flagella 6. Moreover, FSIP2 can bind to A-kinase anchoring protein 4, which in turn plays an important role in the fibrous sheath assembly in sperm flagella. Hence, mutations in *FSIP2* potentially cause loss of binding of A-kinase anchoring protein 4 7. Thus, changes in FSIP2 affect the biological behavior of sperm flagella. Further, whole-exome sequencing revealed that mutations of *FSIP2* affect the development and progression of testicular germ cell tumors 8. Additionally, Lefebvre et al. reported a higher rate of *FSIP2* mutation in metastatic breast cancer compared to early stage breast cancer 9. These findings suggest that FSIP2 is associated with the development and progression of various cancers. However, very few studies have investigated the role of FSIP2 expression in cancer.

In this study, we evaluated FSIP2 expression in 85 patients with clear cell RCC (ccRCC), analyzed the association between FSIP2 expression and clinicopathological characteristics of patients, and correlated expression with patient survival outcomes. Furthermore, we used public databases to explore differential expression and functional significance of FSIP2 in ccRCC.

## Methods and Materials

### Oncomine analysis

The Oncomine database (www.oncomine.org) was used to determine the mRNA levels and copy numbers of *FSIP2* gene in different cancers. Student's t-test was performed to determine if the differences between cancer and normal control tissues were significant. The cut-off value for significant fold-change was set to 2, and that for the *P* value was 0.01.

### Cancer Cell Line Encyclopedia (CCLE) analysis

The CCLE database (https://portals.broadinstitute.org/ccle/home) was used to analyze *FSIP2* mRNA levels in a series of cancers. The DNA copy number and other data types related to methylation, mutation and gene expression were derived from 1457 human cancer cell lines to aid the analysis of genetic, pedigree, and predictive factors influencing drug sensitivity.

### Patients and clinical specimens

Eighty-five randomly selected patients with pathologically confirmed ccRCC were enrolled in this study. Specimens were obtained from the patients during surgery at the China Medical University between January 2013 and October 2015. All the tissue specimens were pathologically confirmed to be ccRCC. The inclusion criteria for the patients were as follows: 18-80 years of age, ccRCC, no distant organ metastasis at the time of surgery, and a 5-year postoperative follow-up evaluation. The exclusion criteria were as follows: a preexisting diagnosis of other cancers, preoperative adjuvant treatment, and lack of clinicopathological data. The protocol for this retrospective study was approved by the China Medical University Ethics Committee. All patients provided informed consent prior to the collection of tissue specimens. The following clinicopathological characteristics of the patients were recorded during the sample collection: age, histological grade, serum Ca^2+^ and hemoglobin levels, neutral granulocyte count, and platelet count.

### Immunohistochemistry analysis

Immunohistochemical staining of the primary renal cell tumor biopsy specimens collected from 85 patients with ccRCC was performed to detect the expression and determine the cellular localization of FSIP2 protein. The specimens were fixed in 4% formaldehyde and embedded in paraffin. The specimens were then sliced into 5 μm thick sections, pretreated with 3-aminopropyltriethoxysilane, deparaffinized, rehydrated, and incubated with a primary rabbit polyclonal anti-FSIP2 antibody (1:150; ab150351, Abcam, Cambridge, UK) at 4 °C overnight followed by an incubation with secondary antibody (Gene Tech Co., Ltd., Shanghai, China) for 1 h, and visualized using DAB (Gene Tech Co., Ltd.). Since the primary FSIP2 antibody, ab150351 is a rabbit polyclonal antibody, normal rabbit serum (T8570, Solarbio, Beijing, China) was used as the isotype control. The samples were examined by two pathologists independently.

FSIP2 expression was evaluated semi-quantitatively as follows: a score of 0 was assigned if < 1% cancer cells expressed nuclear and/or cytoplasmic FSIP2; a score of 1+ was assigned if ≥ 1% and < 10% of cancer cells expressed nuclear and/or cytoplasmic FSIP2; a score of 2+ was assigned if ≥ 10% and < 50% of morphologically unequivocal cancer cells expressed nuclear and/or cytoplasmic FSIP2; a score of 3+ was assigned if ≥ 50% cells expressed nuclear or cytoplasmic FSIP2. The cancer cells with scores of 2+ and 3+ were considered FSIP2-positive.

### Statistical analysis

Disease-free survival (DFS) is the time from surgery/treatment to the recurrence of distant organ metastasis. Overall survival (OS) is defined as the time from surgery to death. The relationship between FSIP2 expression and these clinicopathological characteristics were statistically evaluated using the Chi-squared test and independent sample t-test. The DFS and OS of these patients were analyzed by Kaplan-Meier survival analysis, and differences were assessed using the log-rank test. SPSS software (version 21.0; SPSS Inc., IL, Chicago, USA) was used to perform the statistical analysis. *P* values < 0.05 were considered statistically significant.

## Results

### Levels of *FSIP2* mRNA transcripts in RCC

**Figure 1:**
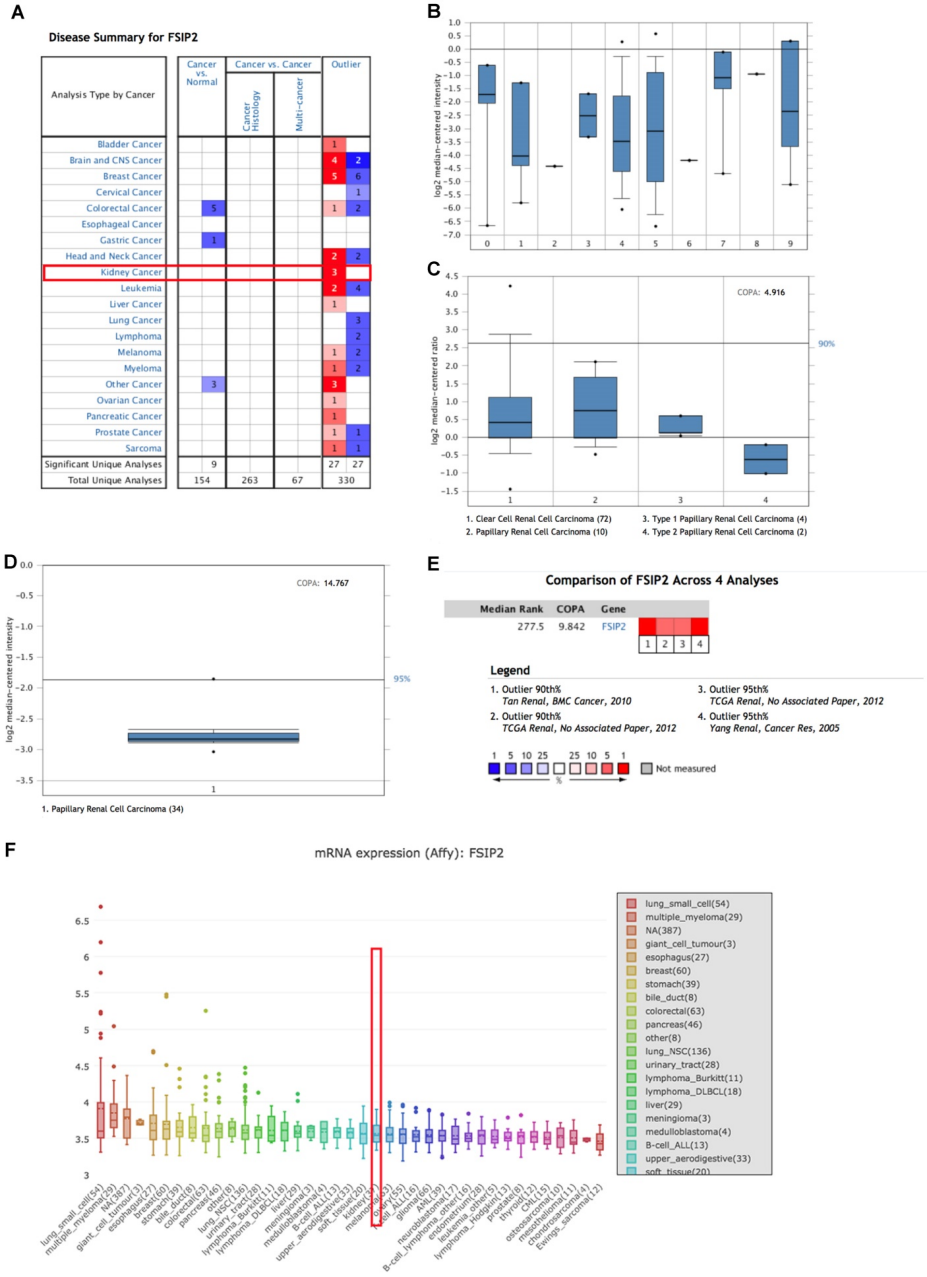
Assessment of *FSIP2* mRNA transcript levels in renal cell carcinoma using public databases. **A:** Levels of *FSIP2* mRNA transcripts in different tumor types obtained from the Oncomine database. **B:** Comparison of *FSIP2* mRNA expression levels from different research groups in Oncomine database (X axis represents the data from different research groups). **C-D:** *FSIP2* outlier analysis using Oncomine database (X axis represents the data from different research groups); numbers in parentheses represent the sample size. **E:** Meta-analysis of multiple datasets to estimate the difference in *FSIP2* mRNA levels between ccRCC and normal tissues using Oncomine database. **F:** *FSIP2* mRNA expression level across various cancer cell lines, including kidney cancer cell lines (rank 22^nd^, indicated by red boxes) from the CCLE database (Y-axis represents the expression level of FSIP2 mRNA in different cancer cell lines).

We explored the levels of *FSIP2* mRNA transcripts in RCC using the Oncomine cancer database. The differential expression of *FSIP2* mRNA was found to be reported in 20 human cancers (**Figure 1A**). However, no result was obtained for FSIP2 expression in the RCC vs normal tissues, as shown in **Figure 1A**. Nevertheless, based on datasets from other research groups, Oncomine analysis revealed that *FSIP2* mRNA levels in RCC tissues did not differ significantly from those in normal tissues (*P* > 0.05; **Figure 1B**). Therefore, we examined FSIP2 expression in different subtypes of RCC. Due to tumor heterogeneity, t-test may not detect significant changes in differential expression; hence, we performed outlier analysis for FSIP2 expression. Interestingly, FSIP2 was found to be significantly upregulated in papillary RCC (COPA = 4.916) in a dataset comprising 88 samples derived from The Cancer Genome Atlas (**Figure 1C**). In another analysis with 34 samples, *FSIP2* mRNA was upregulated in papillary RCC (COPA = 14.767; **Figure 1D**). To reach a more comprehensive conclusion, we performed a meta-analysis of multiple datasets and observed significant differential expression of FSIP2 in papillary RCC (**Figure 1E**). Further, the results from the CCLE database showed that *FSIP2* mRNA expression in the kidney cancer cell lines ranked 22^nd^ among the cell lines from different cancer tissues (**Figure 1F**).

### Relationships between FSIP2 expression and clinicopathological characteristics

A total of 85 surgical ccRCC specimens were analyzed by immunohistochemistry. The correlation between FSIP2 expression and different clinicopathological characteristics is presented in **Table 1**. The results indicate that FSIP2 expression is not significantly correlated with age, histological grade, serum Ca^2+^ and hemoglobin levels, and neutral granulocyte count. However, it is significantly associated with platelet count (*P* = 0.037), distant metastasis (*P* = 0.028), and mortality (*P* = 0.037).

**Figure 2:**
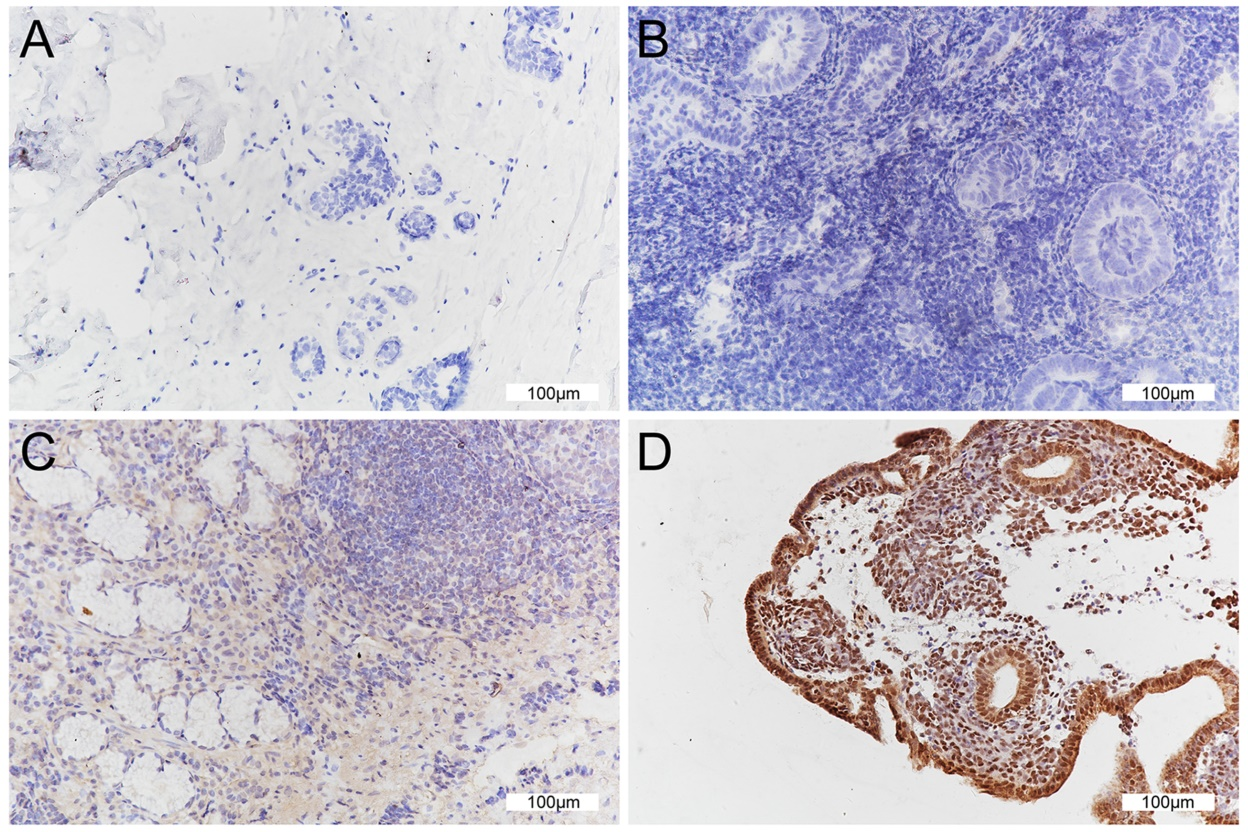
Immunohistochemical analysis of FSIP2 protein in ccRCC patients. **A:** Absence of FSIP2 expression in tumor-adjacent tissue. **B:** Absence of FSIP2 expression in ccRCC tissues. **C:** Low FSIP2 expression in ccRCC tissues. **D:** High FSIP2 expression in ccRCC tissues.

FSIP2 expression was detected in the nucleus and cytoplasm of ccRCC cells in immunohistochemical analysis, while it was not detected in the control samples without primary antibody as shown in **Figure 2**. FSIP2 was expression was observed in 31 patients, of which 19 experienced distant metastases, and 11 out of those 19 (57.9%) were FSIP2-positive (had immunohistochemical score of 2+ or 3+). Furthermore, 15 patients died, including 9 (60%) characterized as FSIP2-positive. Overall, FSIP2 expression levels were higher in RCC patients with distant metastasis than in those without distant metastasis or in the surviving patients **(Figure 2)**.

### FSIP2 expression in ccRCC is associated with worse prognosis and shorter DFS and OS

**Figure 3:**
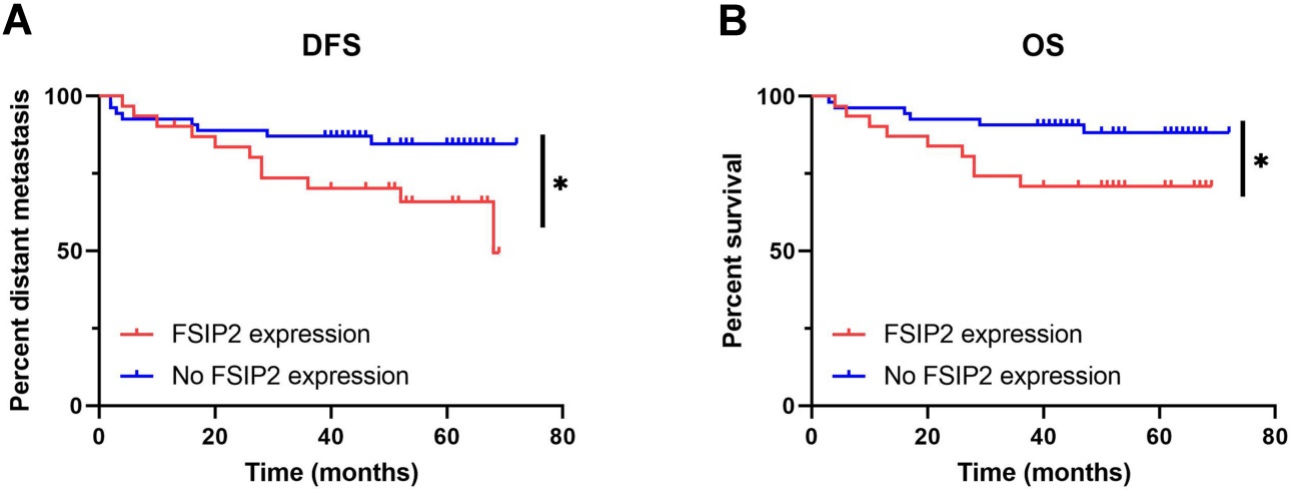
Effect of FSIP2 expression on the survival outcomes of patients with ccRCC. **A:** FSIP2 expression is related to a shorter DFS (*P* = 0.049). **B:** FSIP2 expression is related to a shorter OS (*P* = 0.039).

We also observed that FSIP2 expression is significantly correlated with death (*P* = 0.037) and distant organ metastasis (*P* = 0.028), indicating a role for FSIP2 in adverse prognosis. Hence, we performed survival analysis to explore the correlation between FSIP2 expression and survival outcomes in ccRCC patients. The results revealed that patients with high FSIP2 expression had a poor prognosis, as well as significantly shorter DFS (*P* = 0.049) and OS (*P* = 0.039) as shown in** Figure 3.**

## Discussion

RCC is a heterogenous cancer, and its progression is influenced by various factors 10. Although immunotherapy has been used for the treatment of RCC patients, the associated therapeutic effects have proven to be insufficient in few patients, due to low efficacy and high toxicity 11. Hence, the molecular mechanisms underlying the recurrence and metastasis of RCC in such patients should be further investigated to identify novel therapeutic targets and improve prognosis.

In the present study, FSIP2 expression was shown to be associated with abnormal platelet count, distant metastasis, and mortality. Immunohistochemical examination revealed that FSIP2 is expressed in the nucleus and/or cytoplasm of ccRCC cells. Further, the rate of distant metastasis and death was significantly higher in patients with FSIP2 expression compared to those without FSIP2 expression. Survival analysis revealed that FSIP2 expression is significantly related to a shorter DFS and OS. Furthermore, patients with FSIP2 expression had worse prognosis than those without, indicating that FSIP2 can be used as a predictor of patient prognosis. Gene expression analysis using Oncomine database revealed that although *FSIP2* mRNA levels did not differ significantly between RCC patients and healthy controls, FSIP2 was significantly upregulated in papillary RCC. These results indicate that FSIP2 may contribute to the progression of papillary RCC.

Although previous studies have reported that mutations in *FSIP2* are associated with morphological abnormalities of sperm flagella, progression of testicular germ cell tumors, and development of metastatic breast cancer 6,8,9, this is the first study to report the role of FSIP2 as a predictor of ccRCC prognosis. Moreover, previous studies did not examine the FSIP2 protein expression levels. Our results indicate that FSIP2 expression is a negative predictor of prognosis in patients with ccRCC. Additionally, FSIP2 may play a role in metastasis, tumor invasion, and chemotherapeutic resistance and may be used as a predictive diagnostic biomarker for the prognosis of ccRCC. Further, we explored the proteins interacting with FSIP2 that may be important in RCC growth and progression, using the Search Tool for the Retrieval of Interacting Genes/Proteins (STRING) database. We found that FSIP2 indirectly interacts with galanin (GAL), transmembrane P24 trafficking protein 3 (TMED3), peptide tyrosine (PYY), and neuropeptide Y (NPY) as shown in Supplementary figure 1. Among these, increased expression of GAL has been reported to promote the migration of renal cancer cells, while its knockdown reduces cell migration and invasion. Further, RCC patients with high GAL expression have a shorter DFS 12. Similarly, overexpression of TMED3 has been correlated with poor survival outcomes in ccRCC patients 13. The functional significance of PYY and NPY expression has also been evaluated in RCC tissues 14. These results indicate that the interaction of these proteins with FSIP2 may regulate its biological functions in RCC.

One must note that there are certain limitations of this study. First, this was an exclusively retrospective study with a small sample size. Hence, meta-analyses, and larger randomized controlled trials are required to validate our results. Also, studies with a larger number of ccRCC patients are warranted to further explore the significance of FSIP2 expression on survival outcomes. Second, we did not explore the role of *FSIP2* in the context of the molecular mechanisms associated with ccRCC recurrence and metastasis. Hence, experimental studies exploring the functional significance of FSIP2 in ccRCC are needed. Finally, the FSIP2-antibody used in this study was a polyclonal antibody. Hence, its specificity requires further validation. However, anti FSIP2 monoclonal antibodies will be used in our future studies.

Overall, our study showed that FSIP2 is expressed in ccRCC patients and is associated with poor survival outcomes and prognosis. Therefore, FSIP2 may serve as a potential predictive biomarker for the prognosis of ccRCC.

## Supplementary Material

Supplementary figures and tables.

## Figures and Tables

**Table 1:** Correlation between FSIP2 expression and clinicopathological characteristics

Variables	FSIP2 expression	No FSIP2 expression	*P*-value
No. of patients	31	54	
**Age (year)**			0.800
≤ 65	21	38	
> 65	10	16	
**Histological grade**			0.979
I	23	39	
II	7	13	
III	1	2	
**Serum Ca^2+^ level**			0.691
Normal	17	32	
Abnormal	14	22	
**Hemoglobin level**			0.481
Normal	7	16	
Abnormal	24	38	
**Neutral granulocyte count**			0.154
Normal	10	26	
Abnormal	21	28	
**Platelet count**			0.037
Normal	27	53	
Abnormal	4	1	
**Distant metastasis**			0.028
Yes	11	8	
No	20	46	
**Death**			0.037
Yes	9	6	
No	22	48	

## Abbreviations

CCLE: Cancer Cell Line Encyclopedia
ccRCC: clear cell renal cell carcinoma
DFS: disease-free survival
FSIP2: fibrous sheath interacting protein 2
OS: overall survival
RCC: renal cell carcinoma
RFS: relapse-free survival
STRING: Search Tool for the Retrieval of Interacting Genes/Proteins
