# Supplementary material for: FSIP2 can serve as a predictive biomarker for Clear Cell Renal Cell Carcinoma prognosis
Source: Int J Med Sci. 2020 Oct 8;17(17):2819–25. doi: 10.7150/ijms.48971 (PMC7645329; doi:10.7150/ijms.48971)

Supplementary figure 1

Association of FSIP2 with other regulatory genes explored by STRING database.

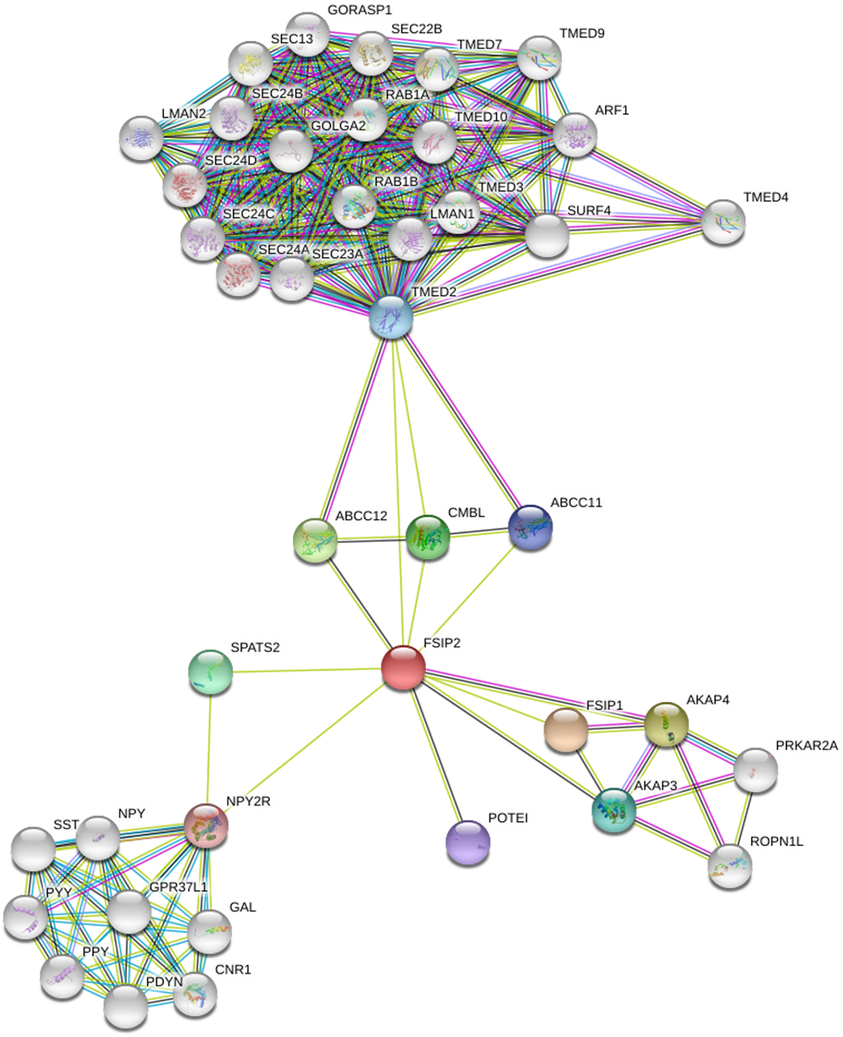

Supplement: Supplementary file 1 — Supplementary figures and tables. [file ijmsv17p2819s1.pdf]
